# Rapid Identification of Hydrogen Isotopes in Water Mixtures by FTIR Spectroscopy

**DOI:** 10.1021/acsomega.5c01180

**Published:** 2025-06-10

**Authors:** Dankun Yang, Norbert Wegrzynowski, Alicja Szczepanska, David Oliver, Keith R. Hallam, Thomas B. Scott

**Affiliations:** Interface Analysis Centre, School of Physics, 1980 University of Bristol, Bristol BS8 1TL, U.K.

## Abstract

A method for rapid determination of the relative concentration of hydrogen isotopes in aqueous solutions was developed based on the rapid measurement of the different molecular bending vibrations using Fourier transform infrared (FTIR) spectroscopy. Isotopic H_2_O and D_2_O mixtures (with D_2_O concentration varying from 0%–100%) were examined in the liquid phase under different environments to estimate the stability, repeatability, and accuracy of FTIR spectroscopy as a rapid assay technique for heavy and light water mixtures. The generated results suggested a high agreeability with the simulated equilibrium constant of the water mixture (average deviation close to 1%) after considering the existence of HDO in the solution. The high sensitivity of the molecular bending motion in detecting the isotopic variations of hydrogen (approximately 0.5%) also proved the reliability of applying FTIR spectroscopy in isotopic determinations. Therefore, we suggested a relatively accurate and rapid identification method for determining the relative proportion of hydrogen isotopes in an aqueous solution by measuring and ratiating the relative peak intensity of the FTIR recorded vibrational peaks.

## Graphical abstract

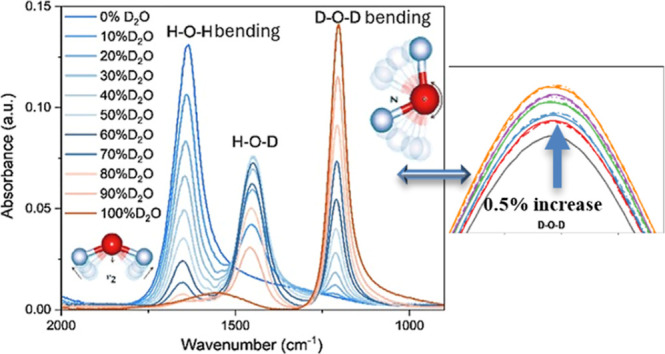

## Introduction

Detection of protium (H) and its heavier isotopes (deuterium (D) and tritium (T)) is required in biological analysis,^1–3^ the nuclear industry,^4–7^ and other isotopic applications^8–10^ routinely. One of the essential reasons for isotopic detection in these areas is to ensure that the deuterium or tritium content in the required sample or solution is quantitatively under control. This is especially so not only for CANDU reactors, which build up T_2_O in the moderator (D_2_O) after a certain period of operation^11,12^ but also for potential fusion fuel storage (D/T mixtures) and waste processing in fusion power plants.^13–16^ This specific requirement of determining the relative abundance of mixed hydrogen isotopes can be achieved by infrared (IR) spectroscopy. Fourier transform infrared (FTIR) spectroscopy identifies the isotopic bonds based on the difference in adsorption wavelength of the vibrational motions. Assuming atoms in the molecules are under harmonic oscillation, the IR adsorption wavenumber of the congeneric chemical bond highly depends on the mass of the atoms comprising the bond, which leads to distinguishable responses between protium, deuterium, and tritium bonds in the compounds.^17–19^ Profiting from that, IR spectroscopy has been applied for deuterium detection in aqueous solutions for decades, particularly for identifying D_2_O in H_2_O solutions.^20–23^ Two different vibrations can be detected and used to determine the isotopic concentration by IR analysis for the H_2_O/D_2_O mixtures: asymmetric stretching of the O–H (3750 cm^–1^ – 2750 cm^–1^)/the O–D (2750 cm^–1^–2000 cm^–1^) and bending (scissoring) of the H–O–H (1850 cm^–1^–1500 cm^–1^)/D–O–D (1250 cm^–1^–1000 cm^–1^)/H–O–D (1500 cm^–1^–1250 cm^–1^).^19,24–26^

Recently, it was noted that using the only bending motion of the water molecules with sharper responses than that of the stretching motions can lead to more accurate results in determining H_2_O/D_2_O compositions as compared to using the stretching motions which sometimes can saturate beyond the maximum limit of detection.^27–30^ However, research into quantitative analysis and simulation of the isotopic composition in H_2_O/D_2_O mixtures using bending motions in FTIR spectroscopy is still limited. Here, we introduce a rapid analytical method for identifying the isotopic composition of isotopically mixed water based on the measurement of the relative intensity of the bending peaks from the absorbance mode of FTIR spectroscopy. The intensities of the adsorption bands in the IR spectrum are determined by the absorbability of the bond, the pathway of IR, and the concentration of the bond in the tested sample. Absorbability is also known as the absolute measure of IR absorbance intensities of the band, while the maximum absorbability can be regarded as the concentration of the specific vibration in the sample.^31–34^ By applying our method, we can simplify the operation and calculation process for determining the isotopic concentration in an H_2_O/D_2_O mixture, thereby offering a rapid means of measuring isotopic change after accounting for the equilibrium kinetics^23^ of hydrogen bonds in the aqueous solution with better accuracy compared to peak-area.^19,26,28^

Here, we will demonstrate how data generated from FTIR spectroscopy show excellent agreement with theoretical calculations of isotopic concentrations in water mixtures under equilibrium conditions, proving that using the maximum intensity of the different bending vibrations can offer a reliable measurement for the isotopic concentration and sensitivity in detecting isotopic variation.

## Experimental Methods

### Sample Preparation

Pure deuterium oxide D_2_O (>99%) and organic standard acetonitrile-d3 CD_3_CN (>99.8%) were acquired from Sigma-Aldrich while pure H_2_O (>99%) used in the experiment was simple deionized water at 18 °C. The mixed isotope solutions were prepared based on a volume-to-volume basis by the same pipet, conducted at room temperature. During preparation of the mixed solutions containing the organic standard, 0.03 mL of CD_3_CN was added to each 1 mL water mixture of different isotopic concentrations. For the FTIR sensitivity test, five different solutions with close concentrations (43.56%, 44.12%, 44.66%, 45.19%, and 45.71%) were analyzed at room temperature.

### Fourier Transform Infrared Spectroscopy

The FTIR spectrometer used in the experiments was a mid-IR spectrometer (4000 cm^–1^–400 cm^–1^; Spectrum Two, PerkinElmer, USA). The molar absorptivity of H_2_O and D_2_O undergoing bending motion is 21.8 ± 0.3 M^–1^cm^–1^ and 17.4 ± 0.2 M^–1^ cm^–1^, respectively, in the mid-IR range at 25 °C, with an optical cell path length of around 3.6 μm for H_2_O and 4.5 μm for D_2_O.^35,36^

For each measurement, a background was taken and removed from the sample analysis; the same amount of 0.02 mL samples was dropped on the detection plate to ensure that the solution fully covered the sample detector with enough IR transmitted through the sample. Experiments were duplicated to ensure the accuracy. Each bond’s (like H–O–H, D–O–D, or H–O–D) bending motion offers an identical response in the same IR source, and the environment of the liquid can cause slight shifting in peak position but the influence of the existence of H–O–D on the intensity of H–O–H and D–O–D is regarded as identical since half of the molecule is from H_2_O and rest of them is from D_2_O.

Spectra were first analyzed using software provided by the FTIR spectrometer supplier (PerkinElmer Spectrum Two), but further data analysis was performed using Origin Lab software. The baselines for the spectra were corrected before normalization and the proportion in 100% D_2_O and 100% H_2_O (0% D_2_O) solution was considered to be 1 and 0, respectively, for subsequent calculations of isotope proportions. By taking pure H_2_O and pure D_2_O as the standard for 100% H_2_O and 0% H_2_O, we can convert the intensity of each H–O–H peak in the series into concentrations and enable comparison of the specific peak H–O–H between the spectra. The proportion calculation for measured HDO was based on the amounts of H_2_O and D_2_O generated from the bending motion of the FTIR spectrum.

## Results and Discussion

### Analysis of a Pure Heavy/Light Water Mixture

When considering an isotopic mixture of H_2_O and D_2_O in pure liquid water, the reaction occurring between H_2_O and D_2_O must be considered, as shown in eq 1. The isotopic solution will undergo mixing and isotopic exchange, reaching an equilibrium state when the reactions become balanced and the quantities of H_2_O, D_2_O, and HDO in the solution are constant.

1 $$H_{2}O+D_{2}O↔2HDO$$

Assuming that H_2_O, D_2_O, and HDO mix ideally in the equilibrated liquid solution, the equilibrium proportion of D_2_O in the ideal liquid mixture of H_2_O can be regarded as

2 $${\left[D_{2}O\right]}_{eq}={\left[D_{2}O\right]}_{in}-\frac{1}{2}{\left[HDO\right]}_{eq}=1-{\left[H_{2}O\right]}_{in}-\frac{1}{2}{\left[HDO\right]}_{eq}$$

where ${\left[D_{2}O\right]}_{in}$ and ${\left[H_{2}O\right]}_{in}$ are the initial amount of D_2_O and H_2_O, respectively, and [HDO]_eq_ is the equilibrium amount of HDO. Similarly, the equilibrium amount of H_2_O can be shown as

3 $${\left[H_{2}O\right]}_{eq}={\left[H_{2}O\right]}_{in}-\frac{1}{2}{\left[HDO\right]}_{eq}$$

The equilibrium constant (*K*_eq_)^23^ of the reaction is considered to be

4 $$K_{eq}={{\left[HDO\right]}^{2}/\left[H_{2}O\right]\left[D_{2}O\right]}_{eq}$$

Combining the results from eqs 2–4, *K*_eq_ can be expressed as

5 $$K_{eq}={{\left[HDO\right]}_{eq}}^{2}/\left[({{\left[H_{2}O\right]}_{in}-\frac{1}{2}{\left[HDO\right]}_{eq})(1-\left[H_{2}O\right]}_{in}-\frac{1}{2}{\left[HDO\right]}_{eq})\right]$$

This leads to an equation with a relationship between the initial fraction of H_2_O and the equilibrium fraction of HDO

6 $$\frac{1}{4}\left(\frac{4}{K_{eq}}-1\right){{\left[HDO\right]}_{eq}}^{2}+\frac{1}{2}{\left[HDO\right]}_{eq}+{{\left[H_{2}O\right]}_{in}}^{2}-{\left[H_{2}O\right]}_{in}=0$$

This equation also applies to the relationship between the equilibrated fraction of HDO and the initial fraction of D_2_O. Therefore, at a defined temperature the fractions of H_2_O, D_2_O, and HDO at equilibrium can be calculated as a function of the initial concentrations of the H_2_O/D_2_O mixture with a known equilibrium constant. The equilibrium constant of the water mixture has been explored with different methods, but the value best derived for H_2_O and D_2_O reactions at 25 °C is 3.86 ± 0.01, generated by Duplan et al. using NMR (nuclear magnetic resonance).^35^ By applying this value of *K*_eq_ to eq 6, the relationship between the initial concentration of D_2_O and the arising equilibrium fractions is as shown in Figure 1a, showing the HDO concentration maximizing at 1:1 D_2_O/H_2_O mixture with a value of around 0.46.

**Figure 1:**
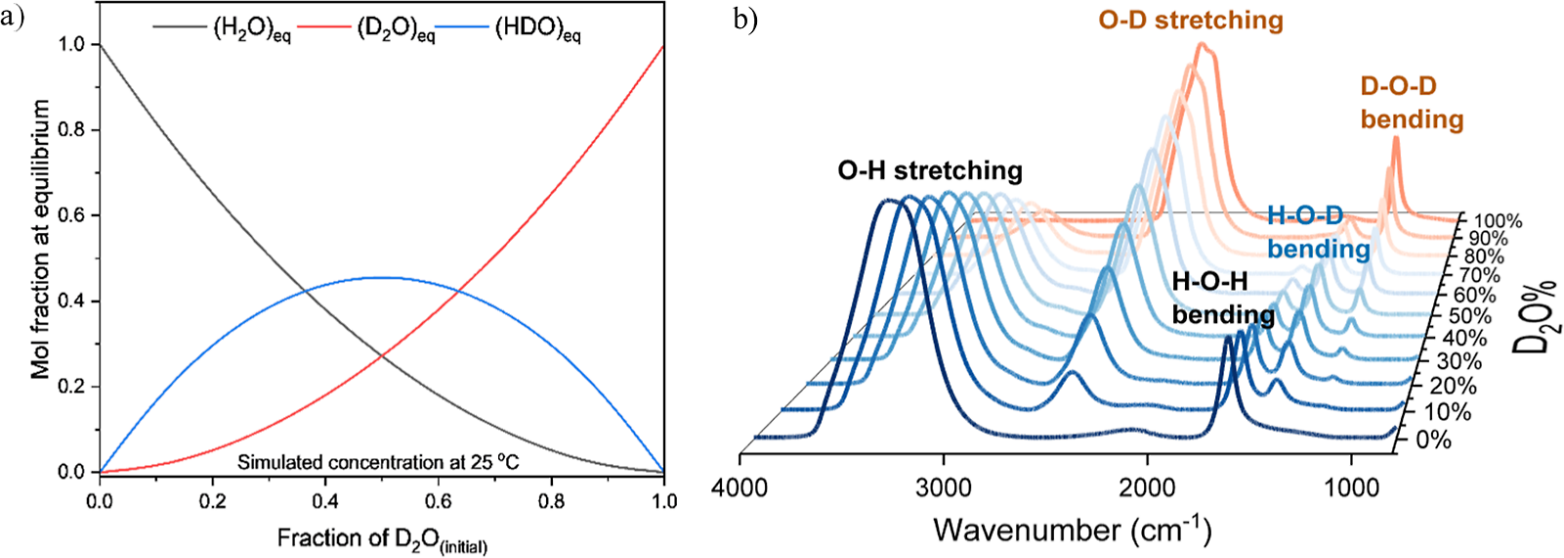
(a) Simulated relationship between H_2_O, D_2_O, and HDO mole fractions and the initial concentration of D_2_O based on eq 6 at room temperature with *K*_eq_ = 3.86. (b) FTIR spectra of pure H_2_O/D_2_O mixtures of varying D_2_O concentrations at room temperature.

This relationship can also be observed in the intensity change of the FTIR HDO bending peak (Figure 1b). When the concentration of an isotope (H or D) increases, the intensities of both the associated stretching and bending vibrations increase correspondingly. However, the intensity of the HDO bending vibration was maximized at 50% concentration of D_2_O/H_2_O mixture, where all isotopic concentrations of protium and deuterium were contributing to the reaction. The position of the generated peaks agreed with the reduced mass calculation and primer literature; both stretching and bending peaks of H_2_O were located at higher wavenumbers than the equivalent D_2_O peaks at a ratio of around 1.37.^28,38,39^ Compared to highly concentrated D_2_O solutions, highly concentrated H_2_O solutions exhibited higher backgrounds in the FTIR spectra, especially at <1000 cm^–1^ (Figure 1b).

As the wavenumber decreases from 4000 cm^–1^ to 400 cm^–1^, the energy (wavelength) of the incident IR wave increases. When the detective bonds in that area (e.g., at wavenumber <1000 cm^–1^) are really limited or do not exist, the IR energy is considered 100% absorbed by the instruments, resulting in a 100% absorbance peak at the very end. This phenomenon affects the background reading even after baseline correction and results in an increase in background noise at a wavenumber below 1000 cm^–1^. Thus, backgrounds were removed before converting the relative peak intensities to calculated mole fraction or proportion.

The mixture proportions were derived from the recorded peak intensities (Figure 2a) of H_2_O, D_2_O, and HDO and suggested an excellent agreement with the theoretical calculation at equilibrium. Limited by the purity of the commercial D_2_O and H_2_O, isotopic impurities can be noticed at notional 100% H_2_O (0% D_2_O) and 100% D_2_O (Figure 2b) as a low-intensity shoulder peak located in the middle of the D_2_O and HDO bending peak range (1500 cm^–1^–1250 cm^–1^) or H_2_O and HDO bending peak range (1700 cm^–1^–1450 cm^–1^), respectively. The intensity shift of the bending peaks can be ascribed to the extremely low or high protium bond environments.^40–42^ These isotopic impurities also led to a nonzero fraction reading (around 1% for D_2_O and 2% for H_2_O) for both 0% and 100% D_2_O sample solutions (Table 1).

**Figure 2:**
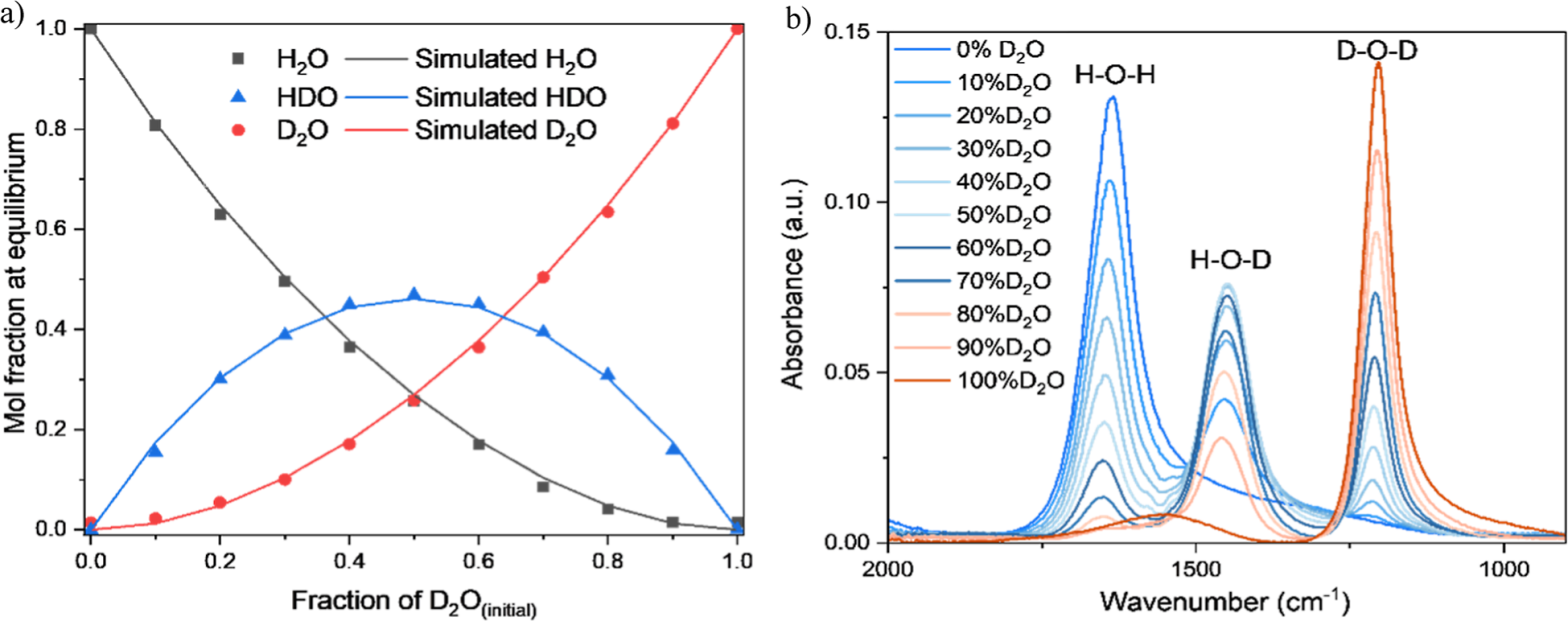
(a) Calculated fractions of H_2_O (red dot), D_2_O (black dot), and HDO (blue dot) at equilibrium, determined from the intensities of the FTIR peaks, and simulated equilibrium fractions of H_2_O, D_2_O, and HDO at room temperature in solid lines. (b) Bending motions of H_2_O, D_2_O, and HDO at different concentrations measured by FTIR spectroscopy at room temperature.

**Table 1:** Initial Fraction of D_2_O and H_2_O before Mixture with a 10% Concentration Increase per Solution, the Equilibrated Fraction of the Mixture Measured by Peak Intensity of Bending Vibration and Simulated Equilibrium Fraction from the Equation at Room Temperature

initial fraction		equilibrium fraction			simulated fraction		
D_2_O	H_2_O	D_2_O	H_2_O	HDO	D_2_O	H_2_O	HDO
0	1.00	0.01	1.00	0	0	1.00	0
0.10	0.90	0.03	0.81	0.15	0.01	0.81	0.17
0.20	0.80	0.06	0.63	0.31	0.05	0.65	0.30
0.30	0.70	0.10	0.50	0.39	0.10	0.50	0.39
0.40	0.60	0.17	0.36	0.45	0.18	0.38	0.44
0.50	0.50	0.26	0.26	0.47	0.27	0.27	0.46
0.60	0.40	0.36	0.17	0.45	0.38	0.17	0.44
0.70	0.30	0.50	0.09	0.40	0.50	0.10	0.39
0.80	0.20	0.64	0.04	0.31	0.65	0.05	0.30
0.90	0.10	0.81	0.02	0.16	0.81	0.01	0.17
1.00	0	1.00	0.02	0.01	1.00	0	0

The average deviation between the measured equilibrium fraction and theoretical value was around 0.01 (0.009 for D_2_O; 0.01 for H_2_O; and 0.01 for HDO) and the maximum deviation was around 0.02. The experiments were duplicated to ensure the reliability of the measurements (Figure S1), with results between runs showing near-identical performance and providing strong evidence that the sensitivity and repeatability demonstrated by FTIR spectroscopy can be used for rapidly and accurately identifying the isotopic composition of pure water mixtures at room temperature. Theoretically, the equilibrium constant of the reaction between H_2_O and D_2_O should be identical from 0 °C–30 °C.^18,23,37^ To determine the viability of using the bending peak intensity from FTIR spectroscopy over different temperatures for detecting the isotopic change of hydrogen in the water mixture, the samples used for the 25 °C experiments were frozen and analyzed by FTIR spectroscopy at 0 °C in the form of ice-water mixtures.

The spectra in Figure 3 showed the high repeatability and thermal stability of detecting isotopic proportions using the bending vibration peak with near-identical responses at 0 and 25 °C (more details in Figure S2). Although the stretching peaks (Figure S3) were relatively identical at different temperatures, the bending peaks still suggested higher stability with thermal variation. Some signal noise fluctuation can be observed at 1650 cm^–1^–1500 cm^–1^ for the 0 °C measurements, potentially due to the thermal impact on the background,^43^ but this did not affect the reading of relative isotope concentration.

**Figure 3:**
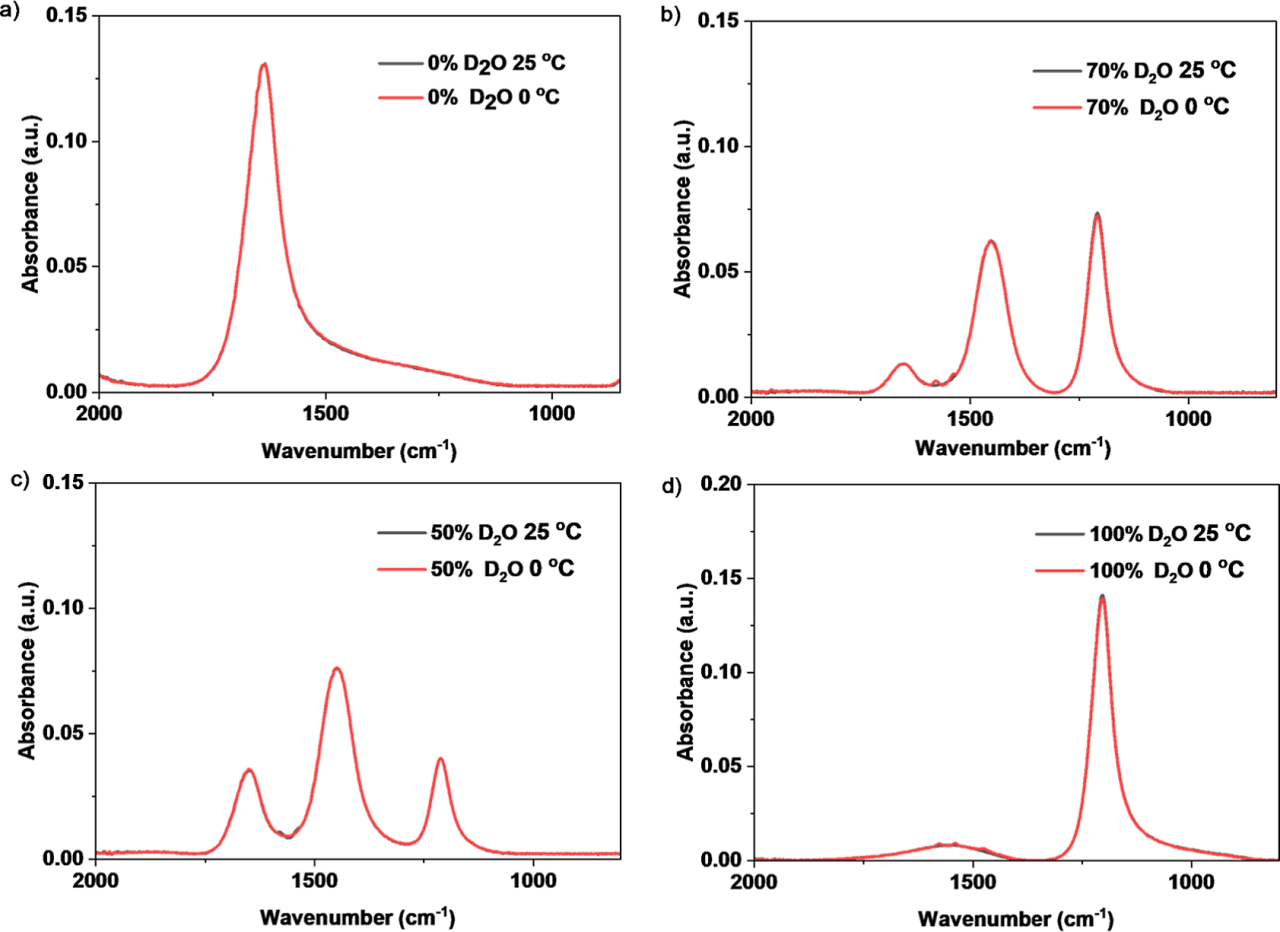
Bending peaks of H_2_O/D_2_O mixtures at different concentrations measured at room temperature and 0 °C.

The reliability of applying bending peak intensity measurements from FTIR spectroscopy in determining the hydrogen isotope proportions was also validated by random testing. In this test, D_2_O solutions with random concentrations (Table 2, fitting results in Figure S4) were prepared and measured at room temperature. The average deviation between the calculated fraction of the water mixture and the simulated fraction at equilibrium was less than 1% (0.007% for D_2_O and H_2_O, 1% for HDO) with a maximum deviation around 2%, agreeing with former experiments. Compared to the fractional determination derived from the HDO peak, the fraction calculated from the bending motion of the H_2_O and D_2_O resulted in less difference versus the theoretical value, indicating that using the peak intensity of the bending vibration for H_2_O or D_2_O can lead to the most accurate reading of the isotopic concentration.

**Table 2:** Initial Fraction of D_2_O and H_2_O at Various Concentrations, Equilibrated Fraction of the Mixture Measured by the Intensity of the Bending Area as Well as the Simulated Equilibrium Fraction from the Equation at Room Temperature

initial fraction		equilibrium fraction			simulated fraction		
D_2_O	H_2_O	D_2_O	H_2_O	HDO	D_2_O	H_2_O	HDO
0.09	0.91	0.02	0.84	0.14	0.01	0.83	0.16
0.21	0.79	0.06	0.64	0.30	0.05	0.63	0.31
0.32	0.68	0.12	0.48	0.40	0.12	0.48	0.41
0.43	0.57	0.21	0.33	0.46	0.20	0.34	0.45
0.54	0.46	0.31	0.22	0.47	0.31	0.23	0.46
0.65	0.35	0.45	0.12	0.43	0.44	0.14	0.42
0.76	0.24	0.61	0.06	0.34	0.59	0.07	0.34
0.87	0.13	0.77	0.02	0.21	0.76	0.02	0.22
0.98	0.02	0.96	0.01	0.03	0.96	0.01	0.04

The close agreement between the calculated isotopic fractions and theoretical data suggests that the function (eq 6) can be used to predict the equilibrated isotopic ratio in any water mixture with the use of FTIR spectroscopy. In other words, once the equilibrium constant of the D_2_O and T_2_O reactions is known, it is possible to simulate the equilibrium fraction of the heavy isotopes in the mixture. Therefore, FTIR can also be applied to the measurement of D_2_O/T_2_O mixtures or H_2_O/T_2_O mixtures because the locations of the vibrational peaks in IR spectra are directly related to the mass of the bond. The peak location of the T_2_O bending vibration should be distinguishable from lighter isotopic bonds,^19,38^ with theoretical wavenumber ratios around 1.58 for the O–T/O–H bond and 1.16 for the O–T/O–D bond which offer a novel way of detecting mixed hydrogen isotopic ratios in water samples.

### Analysis of Hydrogen Isotope Mixtures in an Organic Environment

An investigation of the influence of the solvent environment on the FTIR signal response of hydrogen isotope mixtures was accomplished by adding an organic solvent without free hydrogen or hydroxide groups to the pure water mixture and collecting the equilibrium spectra of the solutions at room temperature. The particular solvent (CD_3_CN) was chosen as it is not considered to be able to react with either D_2_O or H_2_O and is stable at experimental temperatures. By adding the same amount of solvent into the different test solutions, the intensities of the solvent peaks could be controlled. As shown in Figure 4a, the isotopic hydrogen fractions calculated from the peak intensities of the bending vibration still offered high agreement with the theoretical curves. According to the recorded spectra (Figure 4b), the presence of the solvent did not cause an increase in the signal background, especially compared with H_2_O, which potentially reduced the deviation from the theoretical value. Moreover, the location of the vibration peaks from CD_3_CN was not in the measurement range of the bending motions of H_2_O or D_2_O, further reducing any error that adding the solvent may cause. This result suggests that the method can be applied for quantitative analysis of hydrogen isotope ratios in solvent/water mixtures if the solvent has FTIR-observable peak positions suitably distanced from the hydrogen bonds and is stable in aqueous environments.

**Figure 4:**
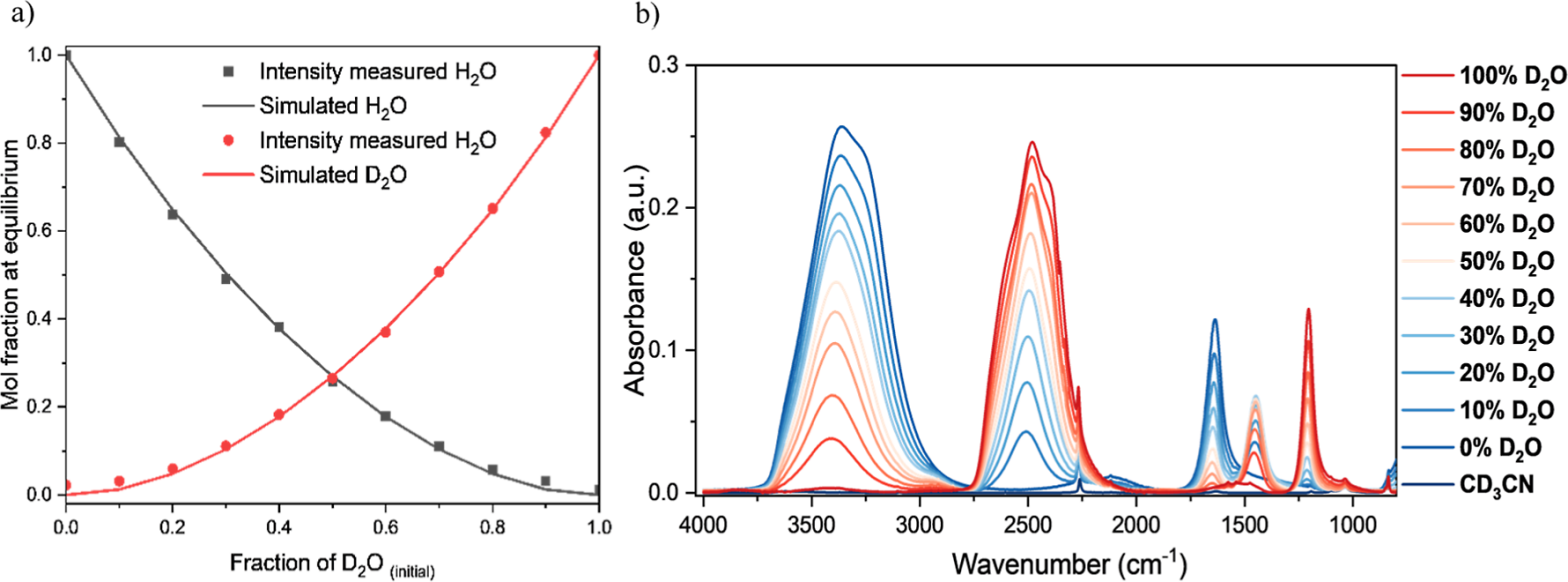
(a) Equilibrium fraction of simulated and measured H_2_O and D_2_O. (b) FTIR absorbance of H_2_O/D_2_O mixtures with CDCN_3_ as a standard solvent in terms of the initial concentration of D_2_O at room temperature.

To reduce the potential influence caused by the infrared waves penetrating through the sample, ATR-FTIR (attenuated total reflectance-FTIR)^44^ was also applied to the test mixture (Figure S5). However, the diamond detector, which is an intrinsic part of the instrument, reduced the signal-to-noise ratio significantly around 2000 cm^–1^ influencing the background reading in the spectra. Moreover, limited by the refractive index of the liquid sample, the IR cannot penetrate through the required volume of liquid D_2_O resulting in a misreading of intensities for the stretching peak. Thus, the research presented here has focused solely on standard FTIR analysis for all of the measurements, and we discount ATR-FTIR as being less efficient for the same measurements.

### Sensitivity of FTIR Analysis for Hydrogen Isotope Verification

To determine the sensitivity and limitations of the FTIR technique in detecting proportional variations of H_2_O/D_2_O mixtures, measurement of five different D_2_O/H_2_O mixtures from 43.56%–45.71% D_2_O (with ∼0.5% isotopic increase per solution) was carried out at room temperature. For each mixture, measurements were made in triplicate to ensure accuracy of the generated spectra, as shown in Figure 5.

**Figure 5:**
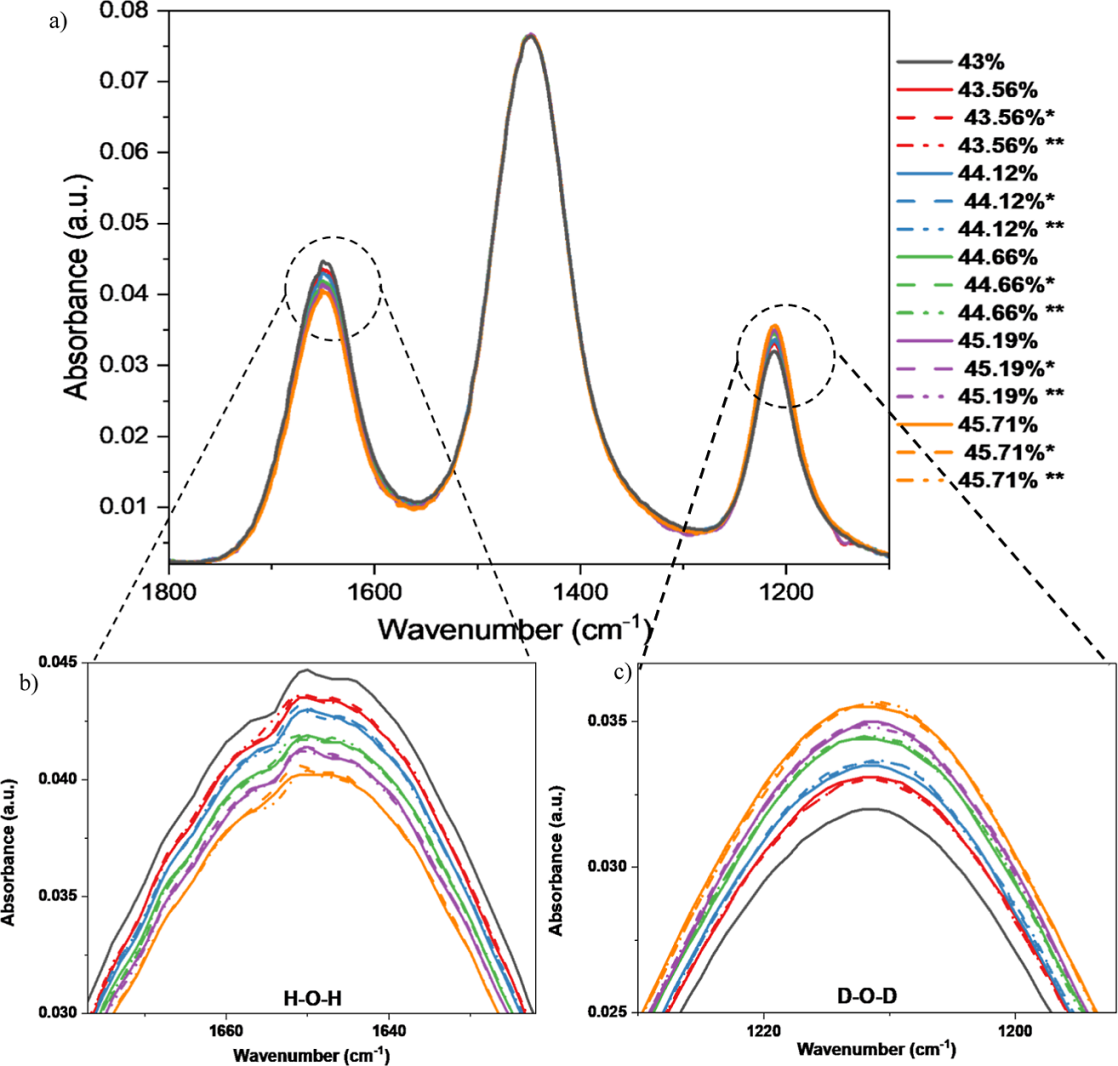
(a) FTIR spectra of 43%–45.71% D_2_O (43% in black, 43.56% in red, 44.12% in blue, 44.66% in green, 45.19% in purple, 45.71% in orange; * stands for the first duplication experiment in dash line, ** stands for the second duplication with dash-dot line). (b) Emphasized H_2_O peak (at around 1650 cm^–1^) of 43%–45.71% D_2_O. (c) Emphasized D_2_O peak (at around 1211 cm^–1^) of 43%–45.71% D_2_O.

The HDO peak shown at 1450 cm^–1^ was identical for all solutions from 43.56%–45.71% D_2_O, indicating that the bending motion for HDO was unsuitable for detecting small variations in hydrogen isotope ratios in the liquid mixture, as also proved in the former experiments (Figure 5a). Unlike the HDO bending vibration, the IR peaks associated with the bending motions of the H_2_O and D_2_O molecules resulted in a clear and measurable intensity difference with changes in the D_2_O concentration between samples (Figure 5b,c). Repeat measurements confirm the stability and reliability of using the intensity change in these vibrational peaks to represent the proportional (isotopic density) increase. Using the equilibrium fraction of the 43% D_2_O solution as a reference, equilibrium fractions of the 43.56%–45.71% D_2_O solutions can be calculated (Table 3) suggesting a good agreement among the measurements. For example, the average calculated equilibrium fractions of D_2_O and H_2_O for 43.56% D_2_O solution were 21.78 ± 0.01% and 33.35 ± 0.04%, respectively.

**Table 3:** Initial Fraction, Simulated Fraction, and Calculated Equilibrium Fraction of 43% D_2_O–45.71% D_2_O Based on the Intensity of the Bending Vibration in FTIR; Δ Stands for Fraction Changes between the Solution

initial fraction		equilibrium fraction				simulated fraction			
D_2_O	H_2_O	D_2_O	ΔD_2_O	H_2_O	ΔH_2_O	D_2_O	ΔD_2_O	H_2_O	ΔH_2_O
43%	**57%**	21.31%		34.07%		**20.35%**		**34.35%**	
43.56%	**56.44%**	21.79%	0.47 ± 0.01%	33.33%	0.70 ± 0.13%	**20.85%**	**0.50%**	**33.73%**	**0.62%**
		21.77%		33.40%					
		21.79%		33.33%					
44.12%	**55.88%**	22.12%	0.37 ± 0.14%	32.74%	0.56 ± 0.07%	**21.35%**	**0.50%**	**33.11%**	**0.62%**
		22.12%		32.71%					
		22.18%		32.77%					
44.66%	**55.34%**	22.65%	0.53 ± 0.06%	32.10%	0.64 ± 0.06%	**21.84%**	**0.49%**	**32.52%**	**0.59%**
		22.65%		32.15%					
		22.71%		32.07%					
45.19%	**54.81%**	23.04%	0.38 ± 0.12%	31.72%	0.55 ± 0.10%	**22.32%**	**0.48%**	**31.94%**	**0.58%**
		22.98%		31.72%					
		22.97%		31.57%					
45.71%	**54.29%**	23.37%	0.47 ± 0.12%	31.08%	0.54 ± 0.18%	**22.79%**	**0.47%**	**31.37%**	**0.57%**
		23.44%		31.03%					
		23.50%		30.99%					

The small deviation generated from the measurements further confirmed the reliability of determining isotopic fractions using the intensities of the water-bending vibrations. As the increase from the initial isotopic concentration leads to a nonlinear response in the equilibrium fraction, the theoretical values were calculated to help determine the ideal increase of the equilibrium fraction with changes in the isotopic concentration. For around a 0.5% isotopic increase in D_2_O, the simulated increase of D_2_O equilibrium fraction agreed well with the measured increase of equilibrated D_2_O in the standard deviation error range, showing that FTIR was sensitive in detecting the isotopic variations (with error less than ±0.13% compared to simulated values).

Although the differences between simulated and equilibrium fractions from FTIR were ±1%, the measurements correctly identified an approximately 0.5% isotopic increase in the solution. The lowest absolute detectable boundary around 0.003 au is to be identified from background noise (approximately from 0.0019 au to 0.0025 au) in absorbance peaks; the verification of the intensity can be confirmed if the difference between two peaks is above 0.002 au, which can be separate from the noise of the instrument. Hence, FTIR spectroscopy can be regarded as being both sensitive and reliable in detecting isotopic variation if the starting isotopic composition in the mixture is already known. Therefore, for applications in the nuclear industry where initial isotopic ratios in D_2_O/H_2_O, D_2_O/T_2_O, or H_2_O/T_2_O mixtures can be predetermined, this method can offer a novel and rapid way of detecting isotopic deviation from a defined starting mixture. Moreover, rapidly detecting and understanding the hydrogen isotope ratio in liquid water can control the risks of potential exposure to radiation caused by the T_2_ gas.

Further development of this technique could seek to develop in-line or in situ FTIR measurement techniques to allow responsive in-plant modification of mixed isotope water mixtures. This work also supports a new concept for storing and adjusting the hydrogen isotopic mixture in the liquid phase. If a water mixture (D_2_O/T_2_O) is applied for storing D/T ahead of using electrolysis to produce DT fuel gas, the loss of T through radioactive decay can be measured and the isotopic compositions can be adjusted by T_2_ bubbling. The decay product of tritium (i.e., helium) would be released as a gas without affecting the purity of the mixture.

## Conclusions

In conclusion, FTIR spectroscopy has been demonstrated as an effective technique for determining the ratios of hydrogen isotopes in water mixtures. FTIR results suggested a close agreement of measurements with theoretical analysis based on equilibrium kinetics. The method was proven reliable in estimating the isotopic ratio from 0 °C up to room temperature under different chemical environments with a relatively low standard deviation (average deviation ≤1%), suitable for industrial application. Benefiting from the sensitivity of the H_2_O and D_2_O bending response in FTIR spectroscopy, it is possible to detect small isotopic variations (0.5%) by converting the measured peak intensity change to the fractional ratio of the aqueous solution. This method offers a rapid and practicable way of detecting hydrogen isotopic variations in water mixtures that would be suitable for research and industrial applications that require in situ isotopic determination. For example, FTIR spectroscopy could be applied in situ to a water-based tritium scrubbing plant (i.e., CANDU) to help measure the accumulation rate of T_2_O in cascades of water bubblers.

## Supplementary Material



## Notes

Duplication experiments of FTIR measurements; full-range IR spectra of water mixture; and ATR-FTIR results (PDF)

## Abbreviations

FTIR: Fourier transform infrared
CD_3_CN: acetonitrile-d3
CANDU: CANada Deuterium Uranium
