# Supplementary material for: Rapid Identification of Hydrogen Isotopes in Water Mixtures by FTIR Spectroscopy
Source: ACS Omega. 2025 Jun 10;10(24):25801–9. doi: 10.1021/acsomega.5c01180 (PMC12198985; doi:10.1021/acsomega.5c01180)
Supplement: Supplementary file 1 [file ao5c01180_si_001.pdf]

Supplementary information

Rapid identification of hydrogen isotopes in water mixtures by FT-IR

Dankun Yang [a]; Norbert Wegrzynowski [a]; Alicja Szczepanska [a]; David Oliver [a]; Keith R Hallam [a]; Thomas B Scott [a]*

a. Interface Analysis Centre, School of Physics, University of Bristol, Bristol, UK, BS8 1TL.

Dankun Yang: anna.yang@bristol.ac.uk Thomas B Scott *t.b.scott@bristol.ac.uk

Duplicate measurements of H_2O/D_2O mixtures using FTIR at different temperatures

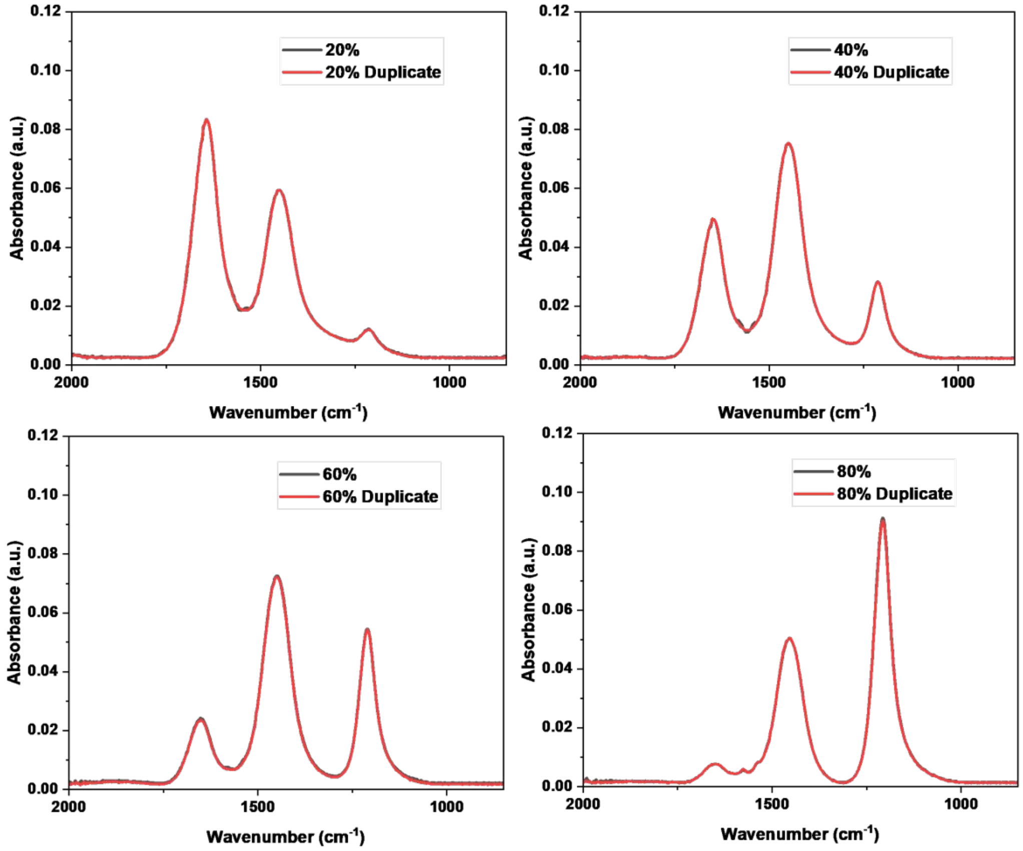

Figure S1: Duplicate FTIR measurements of water mixtures with different concentrations of D_2O at room temperature.

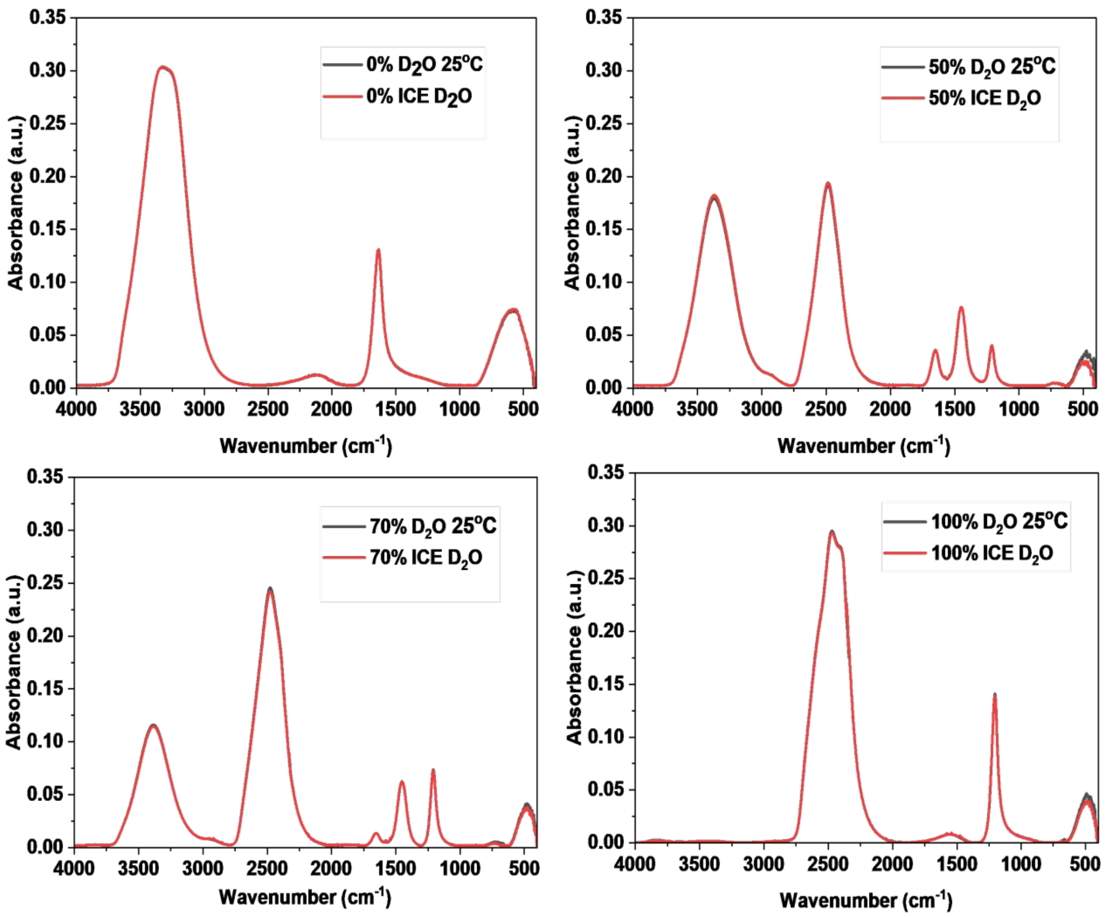

Figure S2: Full-range IR spectra for water mixtures with different concentrations of D_2O tested at 0 °C and 25 °C.

Supplementary information

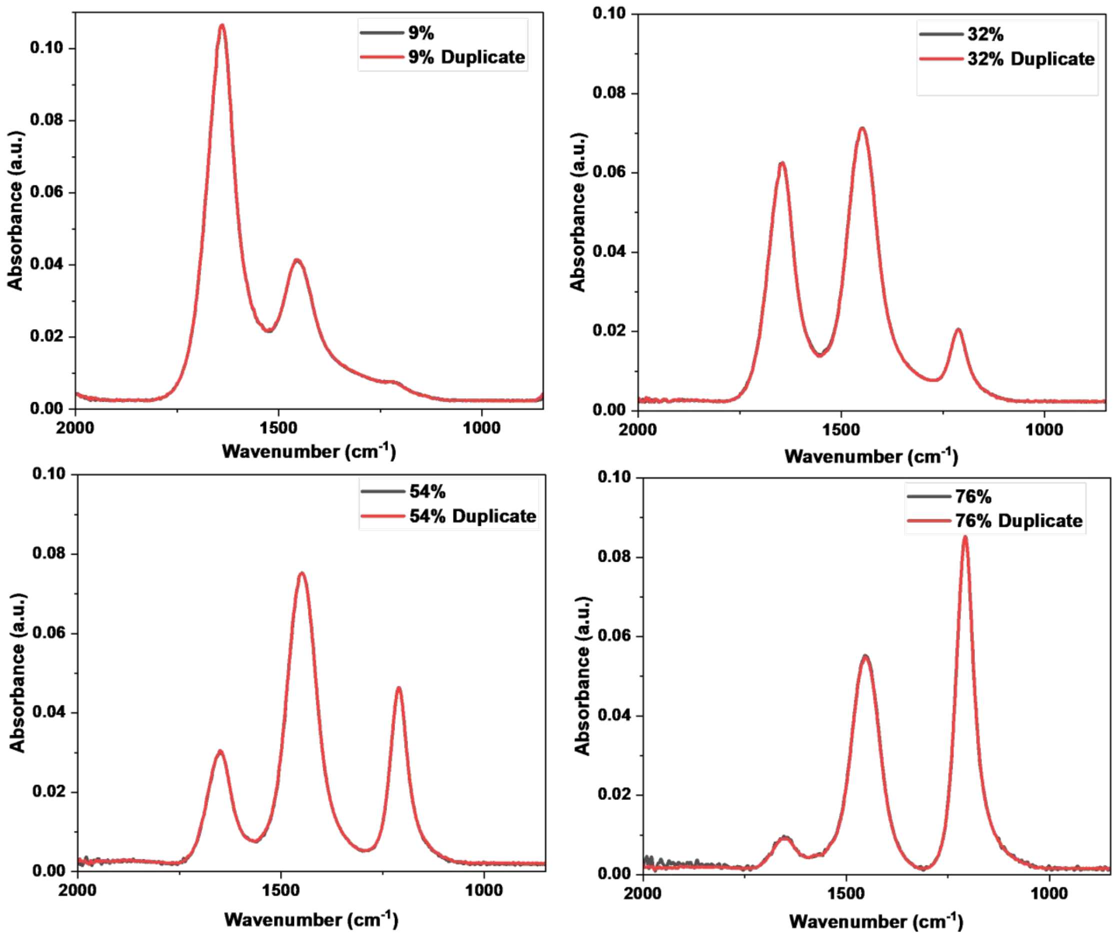

Figure S3: Duplicate FTIR measurements of water mixtures with different concentrations of D_2O at room temperature for blind test.

Fractions generated from various initial concentrationS

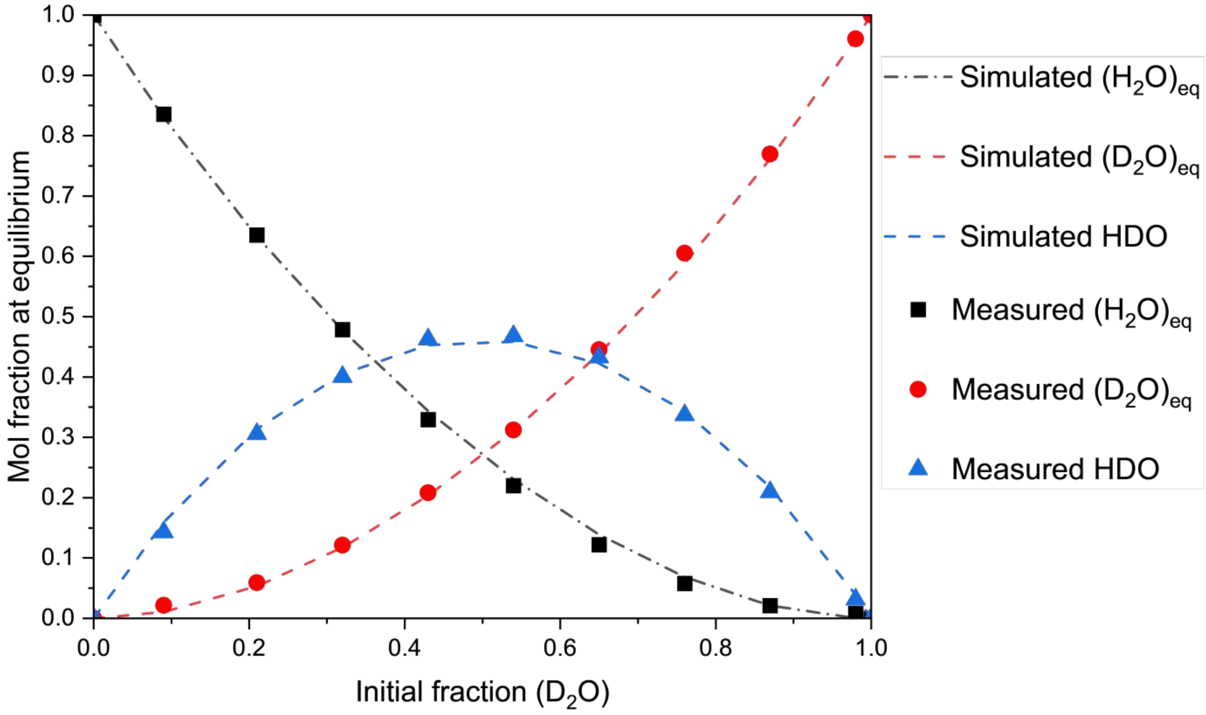

Figure S4: Calculated fractions of H_2O, D_2O and HDO at the equilibrium of random points (9% D_2O up to 98% D_2O) and simulated equilibrium fractions of H_2O, D_2O and HDO at room temperature.

ATR-FTIR measurements of water mixtures in CD_3CN

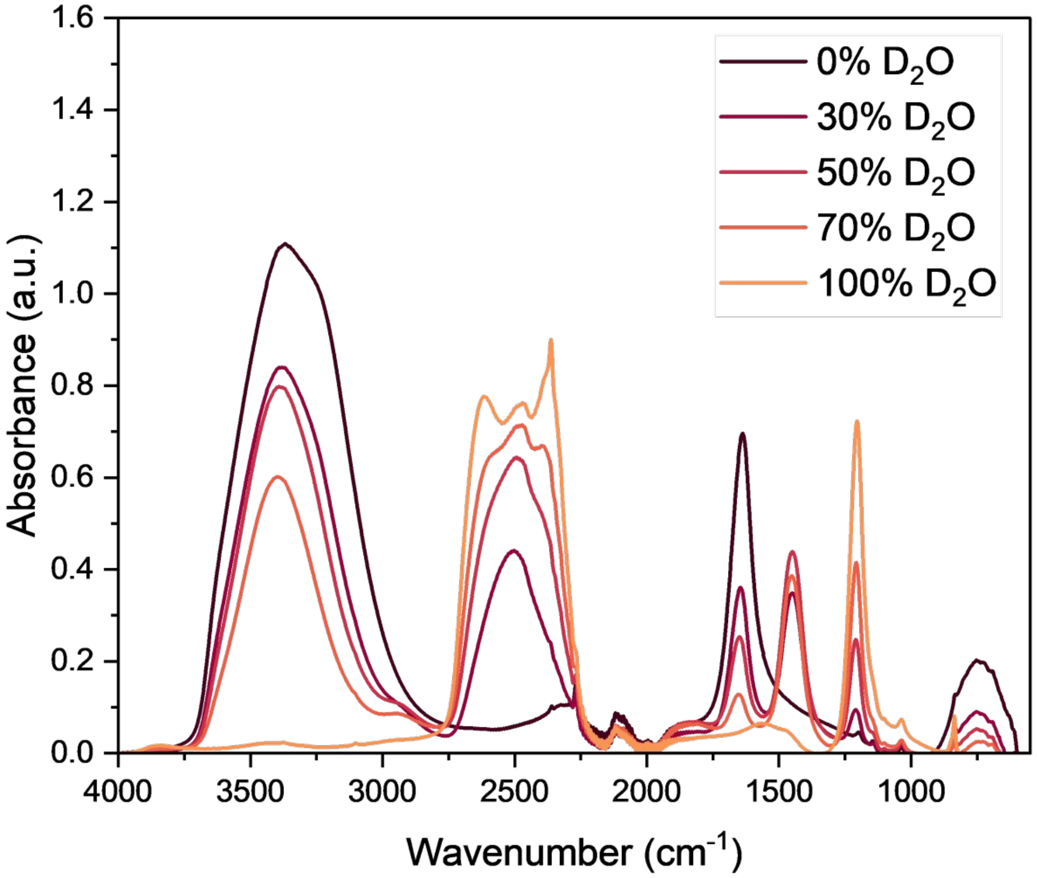

Figure S5: ATR-FTIR measurements of different concentration D_2O/H_2O mixtures in CD_3CN at room temperature.
